# Sublethal interaction factor (SIF), a growth-based method to analyze antibiotic combinations at sub-inhibitory concentrations

**DOI:** 10.1128/spectrum.01070-25

**Published:** 2025-10-20

**Authors:** D. L. Sánchez-Hevia, N. Fatsis-Kavalopoulos, D. I. Andersson

**Affiliations:** 1 Department of Medical Biochemistry and Microbiology, Uppsala University 211745, Uppsala, Sweden; Universidad Maimonides, Buenos Aires, Argentina

**Keywords:** antimicrobial interactions, antibiotic combinations, drug interactions, sub-inhibitory antibiotics, synergy, antagonism

## Abstract

Antibiotic resistance is a global concern with significant implications for healthcare, food production, and the environment. Therapies involving antibiotic combinations are frequently employed as a strategy to overcome antibiotic resistance. When present in combination, the efficacy of antibiotics may be enhanced or weakened, and as antibiotic interactions are *a priori* generally unpredictable, they need to be experimentally determined. Though antibiotics are regularly present at sublethal concentrations (e.g., in patients with suboptimal dosing regimens, late after the last dosage, difficult-to-penetrate tissues, and also in the natural environment), effects of antibiotic combinations are generally studied at lethal dosages. To address this, we developed the sublethal interaction factor (SIF) assay, based on the Bliss independence model, to quantify antibiotic combination effects at sublethal concentrations. SIF assay uses the whole growth curve, instead of only the growth rate, and determines reliably the outcome of the interaction between two antibiotics at sublethal concentrations. The SIF method was validated against the CombiANT assay and showed high sensitivity and specificity, attesting to its usability.

**IMPORTANCE:**

Sublethal interaction factor (SIF), a method herein proposed, simplifies the analysis of antibiotic interactions at sub-inhibitory concentrations and shows a high correlation with the fractional inhibitory concentration index (FICi), the gold-standard measure used to classify antibiotic interactions when tested at lethal concentrations. The SIF can be easily incorporated into laboratories and has great potential for studying not only antibiotic combinations but also drug interactions and phage therapy.

## INTRODUCTION

Antibiotic resistance is a global threat, jeopardizing the efficacy of antibiotics in treating bacterial infections. This, along with the limited number of antibiotics being discovered, has made the use of antibiotic combinations. Antibiotics have also been detected in a wide range of environments (1–7), with several natural habitats identified as reservoirs of antibiotic resistance (7–9). In all those cases, antibiotics are not present individually but in combinations (and in the environment, alongside with the other pollutants). The efficacy of those antibiotic mixtures may range from an effect equivalent to the sum of their individual activities (additivity) to an enhanced effect (synergy) or a reduced effect (antagonism) (10–12). Even though antibiotic combinations are naturally found in the environment, and their use is not new—some date back to the 1950s (13)—in many cases, little is known about the mechanisms underlying the interactions. Many efforts have been made to predict antibiotic interaction effects (14–16), but the species- and strain-dependent nature of antibiotic interaction outcomes (17–21), along with the demonstrated impact of genetic factors on drug interactions (22), reinforces that *in vitro* analyses are still highly relevant.

To date, there is no consensus on the best procedure to quantify antibiotic interactions. Different laboratories not only use different experimental techniques but also different models to analyze the data, such as the Loewe and Bliss models (16, 23–27). The model proposed by Loewe posits that an antibiotic cannot interact with itself, whereas the Bliss independence model assumes that the effect of a drug combination is the product of the single-drug effects. Both Loewe’s and Bliss’ models have limitations (16, 24, 27), and Loewe’s model is the preferred one when analyzing two antibiotics which share a target (22, 28), whereas the Bliss model is used when two compounds act on different targets (12, 16–18, 29, 30).

Several established assays, such as time–kill, checkerboard (28–33) and CombiANT (19, 34–36) assays, are used to study antibiotic interactions, all of which are based on lethal antibiotic concentrations. Time–kill and checkerboard assays are labor-intensive. Meanwhile, the commercial device CombiANT enables higher throughput. Both time–kill and checkerboard assays can quantify the overall growth of the cultures, but the standardized determination is done as an endpoint comparison. For time–kill assays, an increase or decrease of >2log_10_ CFU/mL from the reduction achieved with the most active agent is used to determine whether an interaction is antagonistic or synergistic, respectively (37, 38). In the case of checkerboard and CombiANT assays, the quantification is done using the fractional inhibitory concentration index (FICi; Formula 1) (34, 37–39), considering the MIC (minimal inhibitory concentration) values of each antibiotic tested alone, as well as their inhibitory concentrations (ICs) tested in combination.


(1) $$FICi=\frac{IC_{AB1}}{MIC_{AB1}}+\frac{IC_{AB2}}{MIC_{AB2}}$$

Since a drug cannot interact with itself, a self-drug combination will always be independent (additive), with FICi = 1. Deviations from additivity will entail interactions, with FICi <1 and FICi >1 corresponding to positive and negative interactions, respectively (19, 39). However, in practice, only interactions with FICi ≤0.5 or FICi ≥4.0 are regarded as synergistic or antagonistic, respectively (19, 34, 37, 38).

Despite the widespread use of FICi-based methods, its limitation as a single-point measurement remains a significant drawback since it fails to account for physiological influences during early growth stages, such as the lag phase (40). A more comprehensive approach to capture interaction effects across all growth phases is to calculate the area under the curve (AUC) for different conditions and compare them, providing a more complete assessment of antibiotic interactions. In this sense, some recent works have used the AUC of cfu (AUCFU) as an alternative method to assess the total bacterial burden over the duration of the study (27, 41, 42). However, no definitions for synergy, additivity, and antagonism have been defined for AUCFU (27, 41).

There exist several motivations to determine interactions at sub-inhibitory concentrations (sub-MIC). First, it has been shown that antibiotic resistance can be selected at very low antibiotic concentrations (4, 43–47), and when those interactions are synergistic, they may in fact promote antibiotic resistance evolution since synergy acts as a selective pressure (30, 48). Second, some interactions, such as phage-antibiotics, are generally determined by checkerboards at sub-MIC (49, 50). Moreover, the nature of the drug interaction can often be affected by dosage (51). For sub-MIC assessment, the preferred approach today is the Bliss independence method (S), which compares the exponential growth rate (g) in presence of agents (here AB_1_ and AB_2_) with the control (here noAB; Formula 2) (27, 32). However, as previously noted for FICi, the Bliss independence model is limited by its reliance on a single growth phase—specifically, the exponential phase—potentially overlooking critical physiological effects that occur during other stages of bacterial growth.


(2) $$S=\left(\frac{g_{AB_{1}}}{g_{noAB}}\right)\left(\frac{g_{AB_{2}}}{g_{noAB}}\right)-\left(\frac{g_{AB_{1+2}}}{g_{noAB}}\right)$$

Here, we present the SIF assay, an easy approach for evaluating antibiotic interactions at sub-MIC levels. Unlike the widely used Bliss independence method (12, 17, 29), which relies on exponential growth rate measurements, SIF enables the assessment of sublethal interactions across the whole growth curve. This novel approach helps the understanding of antibiotic interactions.

## RESULTS

**Fig 1:**
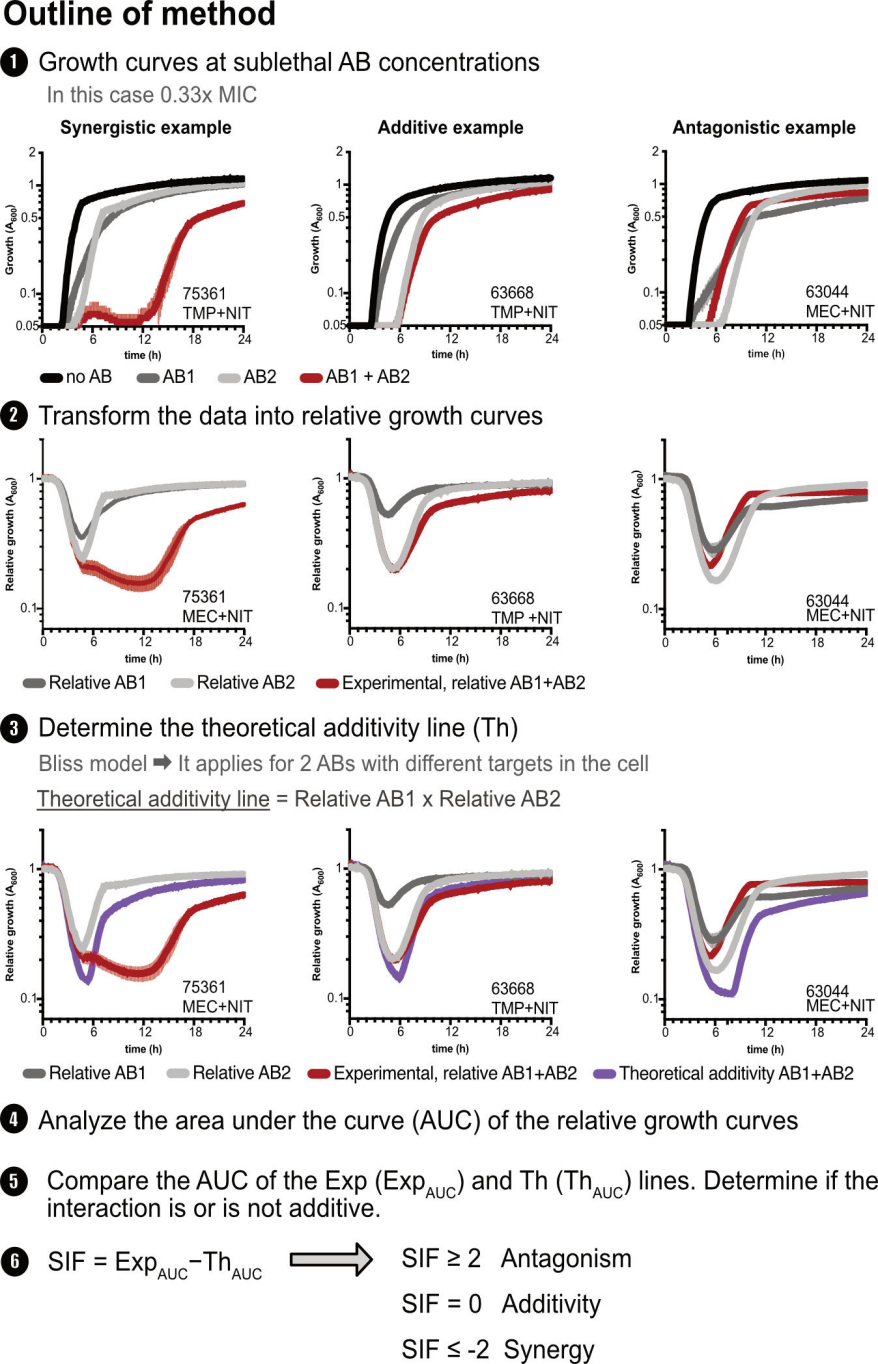
Brief explanation of the determination of antibiotic interaction by SIF. Examples for the three possible outcomes (synergy, additivity, and antagonism) are shown.

SIF is a comparative metric based on the relative growth of bacterial cultures that are exposed to subinhibitory antibiotic concentrations at four conditions: (i) sub-MIC concentration of each antibiotic separately, (ii) sub-MIC concentration of the antibiotic combination, and (iii) a control without antibiotics (from now referred to as noAB; Fig. 1, step 1). SIF is easily performed in microtiter plates, where the four conditions are established in individual wells. The antibiotic concentration used depends on the strain, the antibiotic, and the antibiotic interaction and must be optimized for each case. Each antibiotic must be at a concentration high enough to produce a measurable effect on growth rate, but not so high that the culture is eradicated when both antibiotics are used in combination. Based on our experiments, the best results come from the use of 0.25× to 0.5× MIC dosages.

SIF analysis starts by transforming the complete growth curves into relative growth curves, normalizing to the growth observed in the absence of antibiotics (Formula 3; Fig. 1, step 2).


(3) $$AB_{1}\;relative\;growth\;curve\;=\;\frac{OD_{600}\;AB_{1}\;}{OD_{600}\;noAB}$$

To determine the type of interaction, one compares the relative growth curve in the presence of both antibiotics (from here on referred to as experimental growth or Exp) with the theoretical additivity line or Th (Fig.1, step 3). Th corresponds to the relative growth rate expected at each time point, assuming a Bliss independence interaction (23) (Formula 4, Formula 6). If two antibiotics have a positive interaction, the inhibitory effect is enhanced, and the Exp line will be below the Th. In contrast, when two drugs show a negative interaction, the Exp line is above the Th line (Fig. 1, step 3). Finally, in an additive interaction, the combined inhibitory effect equals the sum of their individual inhibitory effects, and the Exp and Th lines overlap or are nearly identical (Fig. 1, step 3).


(4) $$Theoretical\;additivity\;line=\;\frac{OD_{600}\;AB_{1}\;}{OD_{600}\;noAB}\times \frac{OD_{600}\;AB_{2}}{OD_{600}\;noAB}$$

If one compares two conditions where clearance is observed, as occurs during lag phase, the turbidity difference can be equal to 0 or even negative due to the typical 2% error of the plate readers (suppliers’ information). We propose including a detection limit, such as 0.05 in this example (Formula 5), to avoid negative values that could interfere with the following calculations. This matter has more importance in the Th calculation, where a minimal value is of the utmost relevance. In a hypothetical condition and stage where growth is only detected in plain broth—that is, no growth is observed in any drug condition, either individually or in combination—Formula 4 assumes a repression in drug combination below clearance, which is not physiologically possible. To address this issue, we include the limit of 0.05 in the Th calculation (Formula 6).


(5) $$noABgrowth\;curve\;=\;MAX\left(OD_{noAB}-OD_{blank};0.05\right)$$

(6) $$Theoretical\;additivity\;line=\;MAX\left(\frac{OD_{600}\;AB_{1}\;}{OD_{600}\;noAB}\times \frac{OD_{600}\;AB_{2}}{OD_{600}\;noAB};0.05\right)$$

A mere visual comparison of Exp and Th does not allow a precise quantification of the interaction, and to overcome this issue, one calculates the AUC of the Th and Exp curves (by integrating the respective two plots), thereby converting time curves into a numerical measurement (Fig. 1, step 4). Subsequently, the AUC of the Exp and the AUC of the Th curves are compared using the Wilcoxon signed-rank test (Fig. 1, step 5). If the two measurements differ significantly (*P* < 0.05), then one can conclude that the antibiotics interact, and that the interaction is non-independent. The outcome of the antibiotic interaction can be determined by erasing Th_AUC_ from Exp_AUC_ (Formula 7).


(7) $$SIF=\;Exp_{AUC}-Th_{AUC}$$

To verify that our data were normally distributed, we performed a normality test using 12 independent assays of the DA75361 strain. Both the AUC values of each experimental condition, the additivity theoretical line, and the SIF values passed the tests performed (Table S1; Fig. S1). However, the population size used for validation is extensive, and users of the method are not expected to compile such data, as it is only useful for validation. Hence, we suggest the use of non-parametric tests as part of our analysis that are applicable even in small data sets and remain correct even in normally distributed ones.

**Fig 2:**
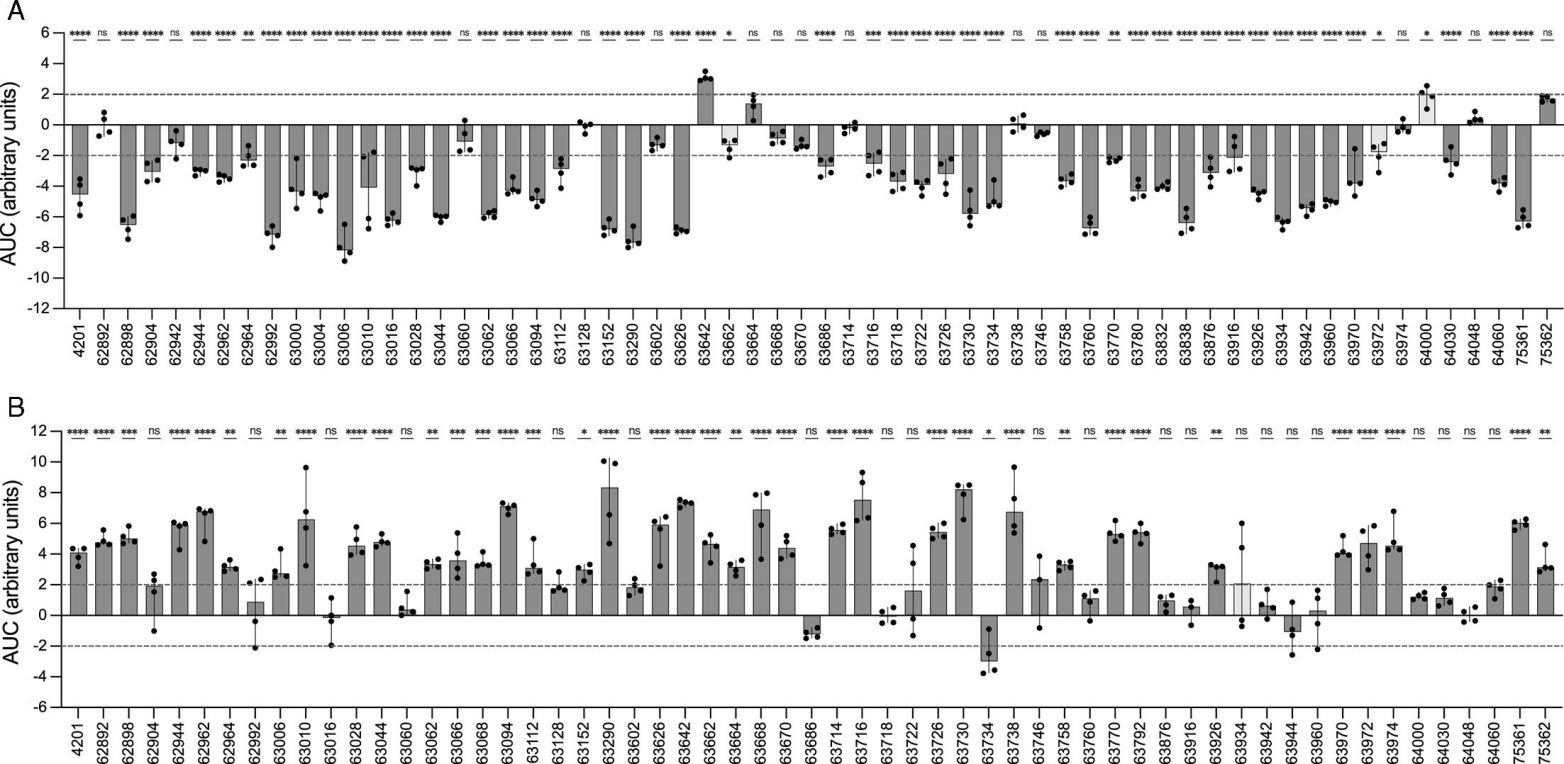
Plot of the SIF result for each of the isolates tested for (**A**) TMP+NIT interaction TMP+NIT (**B**) and for MEC+NIT interaction. The median and the deviation correspond to four independent biological replicates, shown as dots. Statistical analysis indicates whether significant differences were found between experimental (Exp_AUC_) and theoretical (Th_AUC_) AUC values. The cases are categorized differently by SIF, and the statistics are indicated in gray.

Considering that, under independence, the antibiotics do not interact, additivity occurs when SIF = 0, whereas SIF >0 and SIF <0 correspond to negative and positive interactions, respectively (Fig. 1, step 6). Since not all positive or negative interactions necessarily qualify as synergistic or antagonistic, one needs a threshold to define these interactions. To establish such a threshold, we analyzed the statistically significant differences in Exp_AUC_ and Th_AUC_ within our data set. We noticed that interactions classified as synergistic or antagonistic (12, 34) consistently deviated from SIF = 0 by at least a twofold change (Fig. 2). Based on this finding, we recommend using SIF ≥2 to define antagonistic interactions and SIF ≤−2 to define synergistic interactions, ensuring a robust classification framework.

**Fig 3:**
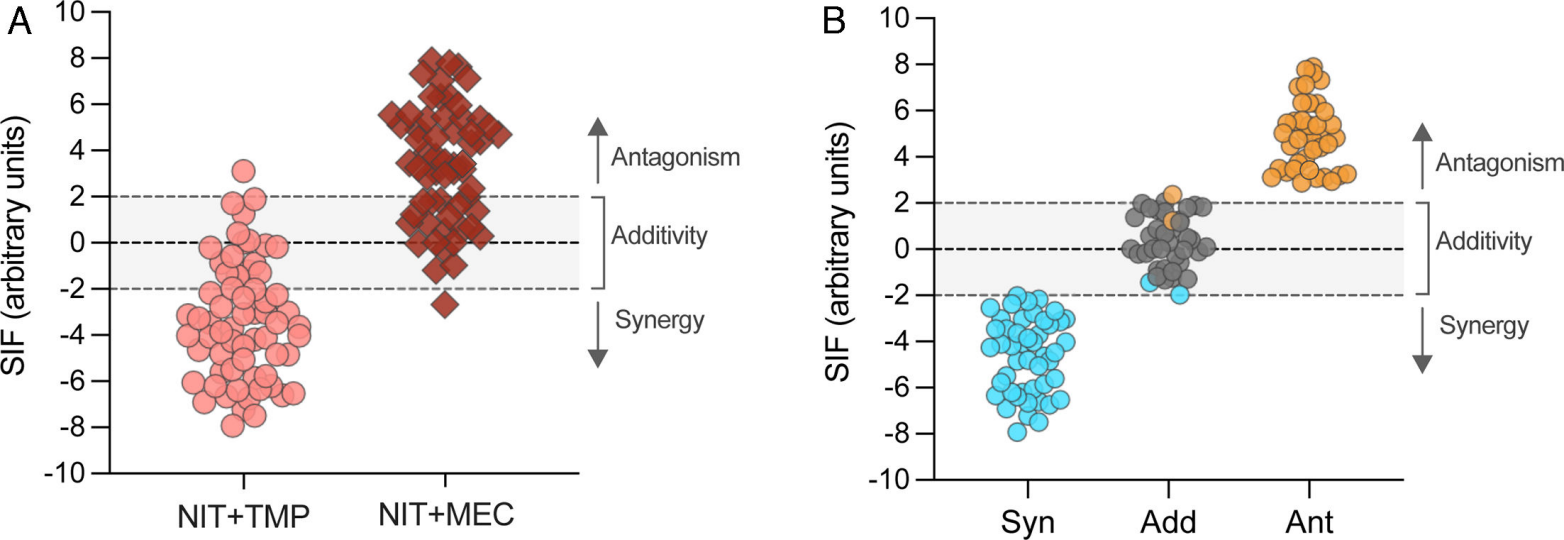
Plot of the SIF result of all the isolates tested, (**A**) according to the antibiotic combination, or (**B**) antagonistic (in orange, ANT), additive (in gray, ADD), or synergistic (in blue, SYNG), according to the SIF value and the significant differences. Only the isolates that showed a SIF value larger than twofold and significant differences between Exp_AUC_ and Th_AUC_ were considered synergistic or antagonistic.

The SIF assay was tested by determining the antibiotic interactions of trimethoprim (TMP) plus nitrofurantoin (NIT) and mecillinam (MEC) plus NIT in a group of clinical *Escherichia coli* isolates. We chose these drugs because they have different modes of action (NIT and MEC are bactericidal and TMP is bacteriostatic) and have been previously described as mainly synergistic and antagonistic, respectively, based on checkerboard and CombiANT assays (12, 34). A total of 121 cases were tested, 62 strains for TMP+NIT and 59 for MEC+NIT combinations. Growth curves were obtained at several sub-MICs, and we selected 0.33× MIC as a favorable concentration to observe a clear effect on growth when each antibiotic acted individually, but not complete growth inhibition when present in mixture. Each case was assessed at the four conditions: (i) without antibiotic (noAB), (ii) only AB1 (TMP or MEC) at 0.33× MIC, (iii) AB2 (NIT) at 0.33×, and (iv) the AB1+AB2 in combination. All analyses and calculations were done as described in Fig. 1. Overall, we found smaller Exp_AUC_ for TMP+NIT (Fig. 3A) and larger Exp_AUC_ for MEC+NIT (Fig. 3B), indicating their synergistic and antagonistic nature, respectively. Those results were confirmed by SIF determination (Fig. 3A), where 68.6% of the isolates showed antibiotic interactions (either synergy or antagonism) and the remaining 28.1% showed additivity, in agreement with the previous work (Fig. 2). Similar differences in antibiotic interaction outcomes among isolates (Fig. 2) were previously described in several works (19–21, 34) and are likely related to isolate-specific genetic differences.

**Fig 4:**
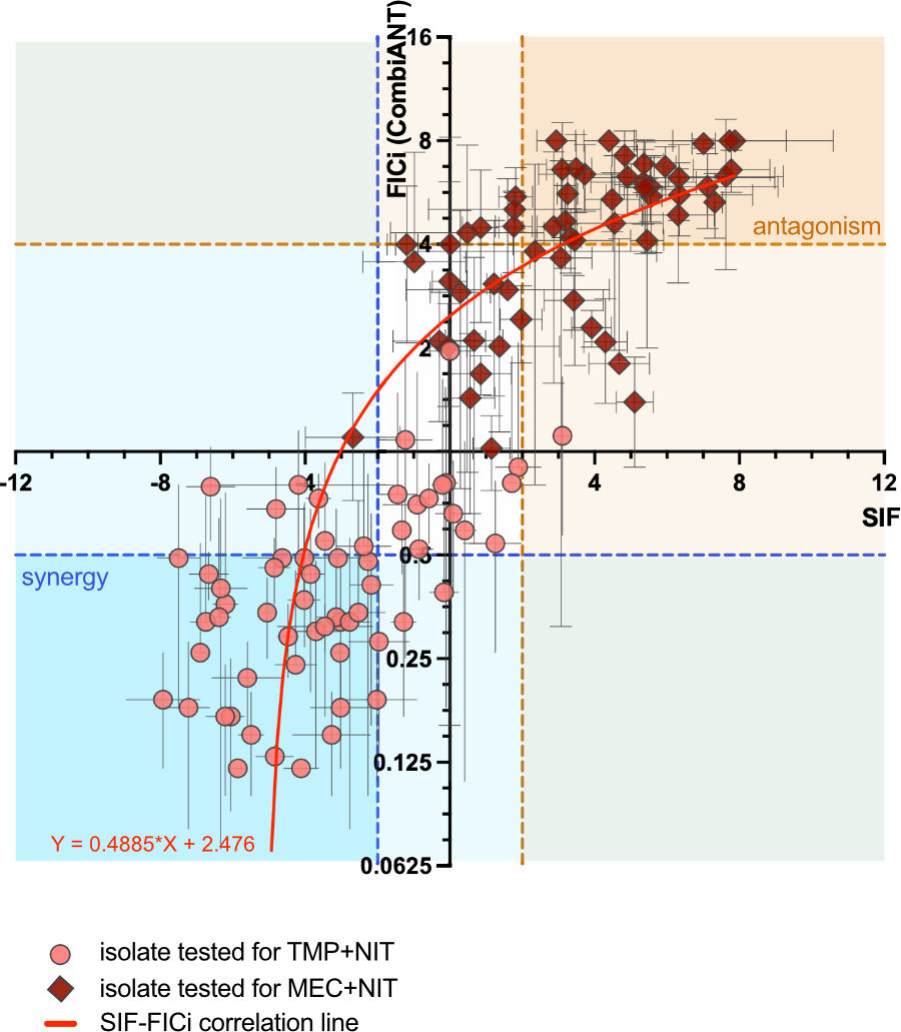
Correlation between SIF and FICi results, showing the average and the standard deviation values of each isolate. The FICi results were analyzed by CombiANT with at least three biological replicates. Both TMP+NIT and MEC+NIT interactions are represented, with circles and diamonds, respectively. The correlation (*P*<0.0001) was verified by a two-tailed correlation test.

To assess the accuracy of the SIF results, we compared them with FICi. We employed CombiANT, a previously validated agar plate-based method to determine FICi (34). We successfully tested 120 out of the 121 original cases. Our results demonstrate that the two classifications consistently produced similar outcomes (Fig. 4), with 80.83% of the cases classified identically by SIF and FICi (Fig. S4). Notably, there were no instances where the two methods assigned conflicting classifications (i.e., one categorizing an interaction as synergistic while the other labeled it as antagonistic). This level of correlation between SIF and CombiANT is equivalent to, or higher than, the correlation previously observed for time–kill and checkerboard (reported as approximately 68–90% in different works) (31, 52, 53), which are among the most used techniques, thereby supporting SIF as a reliable method to explore drug combinations at sublethal doses.

SIF evaluates each case using two criteria: (i) a statistically significant difference between Exp_AUC_ and Th_AUC_ to classify a case as an interaction and (ii) a ≥2-fold change in SIF value to define the type of the interaction. Most of the cases tested here fulfilled both criteria (96.7%), with four of them (3.3%) only fulfilling the first but not the second criterion (Fig. S2; Table S1). A closer look at these discrepancies reveals that all of them correspond to cases with a SIF smaller than twofold, whose variation in AUC was found statistically significant (Fig. 3B). Such results are to be interpreted as an additive interaction with a significant statistical confidence. When compared to the FICi results for the same strains, specificity was found to be 90.6% and sensitivity 85.0% (Fig. S5).

## DISCUSSION

Understanding the efficacy and selection dynamics of antibiotic combinations under conditions where complete bacterial growth inhibition is not achieved is important not only in environment settings, where low antibiotic concentrations are well-documented (1, 3, 4), but also in clinical settings. A single drug can act differently at high and low concentrations, according to the concept of hormesis (54). During treatment, antibiotic levels in the body can drop below the MIC due to factors such as suboptimal dosing regimens or poor patient compliance, underscoring the importance of studying sublethal antibiotic interactions (55–58) and the ability of antibiotics to resist detoxifying enzymes (59). To facilitate measurements of antibiotic interactions at sub-MIC, we developed the SIF method.

SIF classified antibiotic interactions consistently equivalent to FICi, demonstrating its reliability as an alternative method to estimate drug interactions. Moreover, SIF applies strict criteria for assessing an interaction as non-independence: only those pairings that exhibit a statistically significant difference between Exp_AUC_ and Th_AUC_, and have a SIF value exceeding the defined threshold, are classified as true interactions (i.e., non-additive or independent), whether synergistic or antagonistic (Table S1; Fig. 3B). The high accuracy of SIF (80.8%; Fig. S5) is reflected in both the true-positive rate (or sensitivity; 85.0%), and true-negative rate (specificity; 90.6%). We cannot exclude the possibility that those cases with disagreement between SIF and FICi can be related to changes in the action of one of the drugs, as the hormesis concept proposes (54).

SIF determines antibiotic interactions at concentrations causing only partial growth inhibition, integrating effects across all growth stages through AUC analysis. Other traditional methods based on the estimation of the exponential growth rates may fail in detecting the effects on the lag phase or the final yield, and their analysis becomes complicated when a bacterial strain shows a non-sigmoidal growth curve in the presence of antibiotic, as observed for some β-lactams (i.e., MEC) which show pre-lytic OD increase (60). SIF overcomes these limitations by evaluating the effect of drug combinations over the whole growth curve, achieving a more complete assessment of the interactions. As demonstrated here with MEC, SIF is suitable for antibiotics that affect the exponential phase or disrupt the normal sigmoidal growth pattern. Additionally, unlike previous analyses based on AUCFU (27, 41, 42), SIF integrates growth over the entire curve while incorporating well-defined boundaries for different antibiotic combination outcomes. Consequently, SIF refines previous approaches to assessing drug combinations by unifying the analytical power of the AUC-based methods with a more precise characterization of combination outcomes.

The versatility of SIF to assess bactericidal-bacteriostatic and bactericidal-bactericidal combinations, even in the presence of phase-dependent antibiotics, is clear, but since only a few antibiotic combinations have been tested, the generalization of our results to other antibiotic combinations needs further work.

Important advances have been made in understanding the impact of sub-MIC antibiotic concentrations, for example, their ability to activate transcription of certain genes (54) and to confer selection of resistant mutants at concentrations as low as 1/2,000 of the MIC of the susceptible strain for some antibiotics (43–45, 48). However, the impact of this low concentration in drug combinations remains unclear. What is more, the study of drug interactions at sub-MIC is also crucial from both ecological and evolutionary perspectives. Even if synergy at sub-MIC does not completely inhibit bacterial growth, it can still accelerate the rate of evolution of resistance (30, 48). Therefore, the influence of diverse antibiotics, as well as other pollutants such as metals and antibacterial biocides, on bacterial populations in natural habitats remains an active area of research (61, 62). Furthermore, antibiotic susceptibility can be influenced by changes in carbon sources and the availability of micronutrients (i.e., iron) (63–65)—as reflected in the higher production of ß-lactamases in rich medium compared to minimal medium (66)—or by the activation of stress responses (47, 66–68). However, how these conditions alter the antibiotic interactions is not well explored, and SIF makes it simple to explore and adapt different growth conditions effectively.

Herein, we present SIF, a simple and accessible method for characterizing drug combinations that requires only standard laboratory equipment, making it adaptable for microbiology laboratories of all scales. SIF captures interaction dynamics across the full bacterial growth curve, offering a robust alternative to traditional single-point assays. Finally, its versatility extends beyond antibiotics, offering a framework for studying phage therapy, normally tested at sub-MIC, as well as the combined effects of other microbicides, including heavy metals, biocides, and antimicrobial peptides.

## MATERIALS AND METHODS

### Bacterial strains and culture media

The *E. coli* strains used in this work are K-12 MG1665 (referred to as 4201) and bloodstream infection isolates (69). Bacteria were cultivated either in Mueller-Hinton broth (MHB; Becton Dickinson) or on Mueller-Hinton agar (MHA; Becton Dickinson) and supplemented with antibiotics when indicated. The stocks of the used antibiotics—mecillinam (MEC), trimethoprim (TMP), and nitrofurantoin (NIT)—were prepared following supplier’s recommendations (Sigma) using dimethyl sulfoxide (Sigma) or deionized water (Sigma). Bacterial cultures were incubated at 37°C, and growth was monitored by measuring OD_600_. MIC determination was done with E-test strips (Biomerieux) and validated using broth microdilution assay (BMD) (35, 70) for several strains. Equivalent MIC values were obtained.

### Antibiotics interaction determination by CombiANT

FICi and the interaction profile were quantified using CombiANT (Rx Dynamics AB, Uppsala, Sweden), following the previously described protocol (19, 34). At least three independent biological replicates were tested. The FICi was calculated using Formula 1, and the average of all the replicates was determined.

### Sublethal interaction factor (SIF)

The SIF assay compares the growth of the cultures at four conditions: broth (noAB), medium with one antibiotic (either AB1 or AB2), or the two antibiotics in combination (AB_1_+AB_2_). Antibiotics are supplemented at sub-MIC, typically between 0.25× and 0.5× MIC dosages. Here, MEC, TMP, and NIT were supplemented at 0.33× MIC. Each isolate was tested with a minimum of four independent biological replicates. The overnight cultures were diluted in PBS, and 10^5^ cfus were inoculated in each well. The assay was performed in honeycomb round-bottom Bioscreen plates (Bioscreen Inc.). The plates were incubated at 37°C in the Bioscreen (Bioscreen C; FP-1100-C), and the OD_600_ was monitored every 4 min for 24 h. Equivalent results were obtained when using less frequent data points (every 12 or 20 min; Fig. S6).

The analysis of the interaction was done as follows (Fig. 1). First, the growth curves were transformed into relative growth curves by dividing the turbidity in the presence of AB_1_ and/or AB_2_ to the growth of the culture at plain media (Formula 3). Then, following the Bliss model (23), the theoretical additivity line (Th) was determined (Formula 4), which will work as antibiotic interaction threshold.

One further calculates the AUC of the experimental relative growth (Exp_AUC_) and the theoretical additivity line (Th_AUC_). The analysis of the AUC was done in linear scale, and a trapezoid integration method was used. Time point 0 was excluded as relative survival rates do not apply in the beginning of the experiment.

Subsequently, the paired *t*-test was applied to determine differences between Exp_AUC_ and Th_AUC_. Cases deemed statistically significant are associated with deviations from independence; that is, AB1 and AB2 interact, irrespective of whether the interaction is positive or negative. To further assess the type of interaction, one determines the SIF value (Formula 7).

### Statistical analyses

Statistical analyses were performed using Graph Pad Prism; ns indicates non-significant, *P < 0.03, **P < 0.002, ***P < 0.0002, and ***P < 0.0001 for all hypothesis tested in this work.

## SUPPLEMENTAL MATERIAL

Supplemental figures spectrum.01070-25-s0001.docx Figures S1 to S6.

Supplemental tables spectrum.01070-25-s0002.pdf Tables S1 and S2.

ASM does not own the copyrights to Supplemental Material that may be linked to, or accessed through, an article. The authors have granted ASM a non-exclusive, world-wide license to publish the Supplemental Material files. Please contact the corresponding author directly for reuse.
