# Supplementary material for: Sublethal interaction factor (SIF), a growth-based method to analyze antibiotic combinations at sub-inhibitory concentrations
Source: Microbiol Spectr. 2025 Oct 20;13(12):e01070-25. doi: 10.1128/spectrum.01070-25 (PMC12673930; doi:10.1128/spectrum.01070-25)
Supplement: Supplemental figures — Figures S1 to S6. [file spectrum.01070-25-s0001.docx]

Supplementary figure S1: Normal Q-Q plot of the 5 conditions analysed by the normalized test (a) NIT_AUC_, (b) TMP_AUC_, (c) the experimental growth in combination of the two drugs, Exp_AUC_ (d) and the theoretical additivity line, Th_AUC_ (e) SIF. The five conditions passed the normality test.

Supplementary figure S2: 121 isolate antibiotic combinations were assigned as interactions (either synergy or antagonism) or non-interactions (additive) according to the Exp_AUC_ and Th_AUC_ non-parametric t-test comparison and to SIF. Most of them were correspondingly classified as interactions (68.6%) or non-interactions (28.1%) with the two criteria, and only 4 of them (3.3 %) showed a mismatched classification.

(a) (b)

Supplementary figure S3: The AUC of the experimental relative line (Exp_AUC_), in purple, and of the theoretical additivity line (Th_AUC_), in dark red, are indicated for each isolate. The median values were determined from four biological replicates, marked with circles. Results obtained for the (a) TMP+NIT interaction, and (b) MEC+NIT interaction. Significant differences between the AUC of Exp and Th lines were determined using nonparametric paired t-tests.

1.
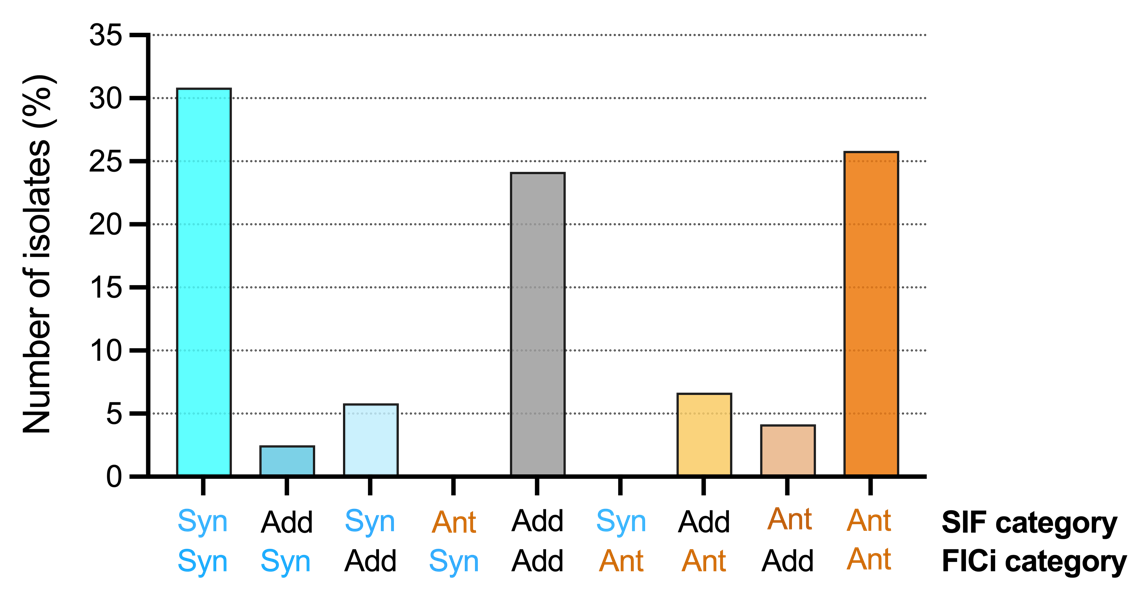


(b)

Supplementary figure S4: Comparison of the distribution by categories of the isolates according to SIF and FICi. (a) Overall, there is an agreement in the classification as synergistic (in blue), additive (in grey) or antagonistic (in orange) when each case is evaluated at lethal (using FICi) or sub-MIC (with SIF) conditions. (b) 80.83% isolates showed the same outcome with SIF and FICi (30,83% synergy, 24.17% additive and 25.83% antagonism). Among the 120 isolates tested, none of them showed an opposite phenotype when the two methodologies were compared.

|  |  | FICi | | | $Sensitivity=\frac{68}{80}\times100=85.0\%$ ${Specificity}_{SYN}=\frac{73}{80}\times100=91.25 \%$ ${Specificity}_{ADD}=\frac{68}{79}\times100=86.08 \%$ ${Specificity}_{ANT}=\frac{76}{81}\times100=93.83 \%$ $Accuracy=\frac{97}{120}\times100=80.83 \%$ |
| --- | --- | --- | --- | --- | --- |
|  |  | Syn | Add | Ant |  |
| SIF | Syn | 37 | 7 | 0 |  |
|  | Add | 3 | 29 | 8 |  |
|  | Ant | 0 | 5 | 31 |  |

Supplementary figure S5: Determination of the sensitivity and specificity of SIF, using FICi values as true classification. The sensitivity of SIF is 85.0 %, whereas the specificity was found to depend on the class, with an average of 90.6 %. The accuracy of SIF is 80.8 %.


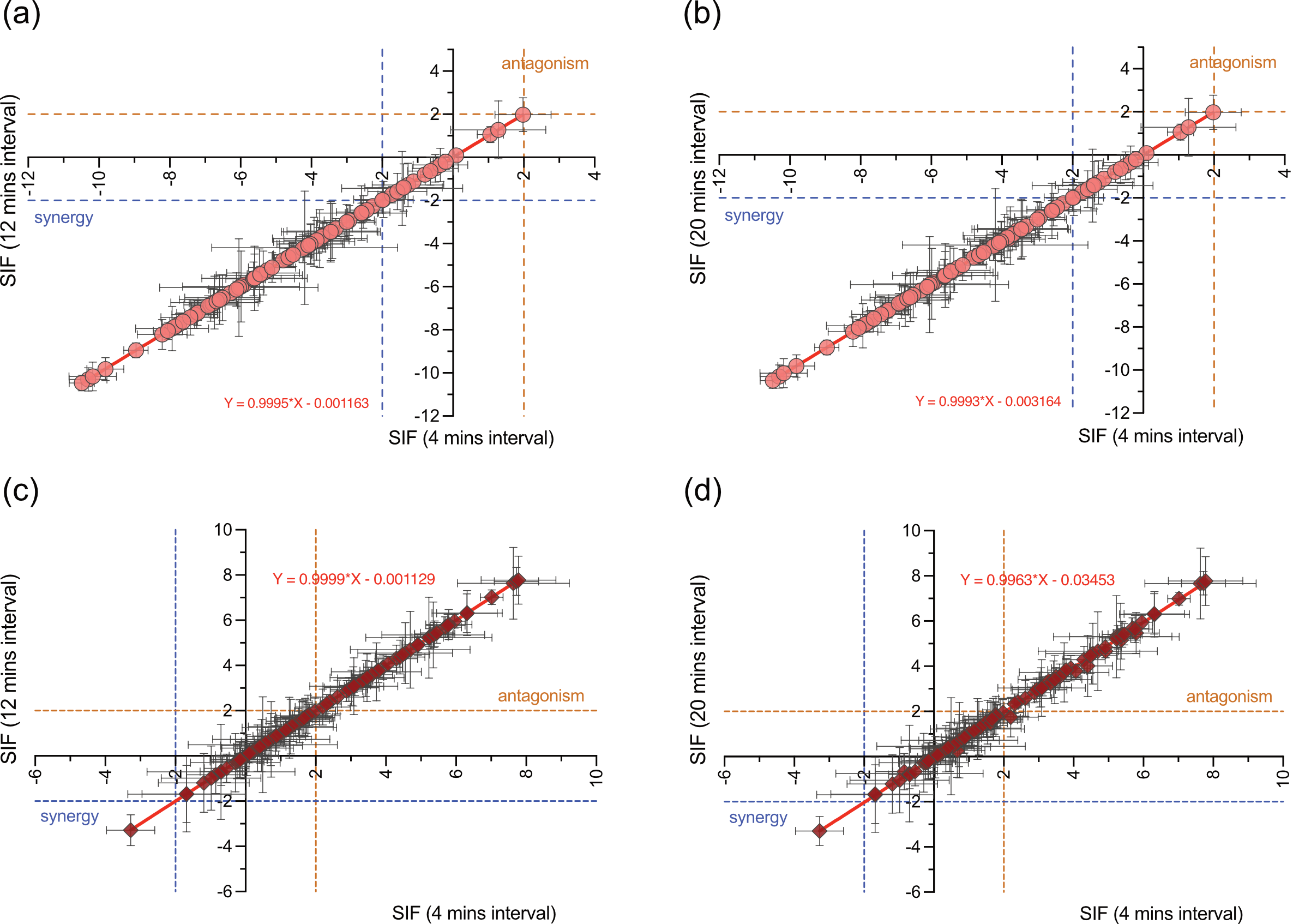


Supplementary figure S6: Linear correlation between the SIF values obtained when using growth curves with 4 minutes measurement interval or less frequent intervals of (a, c) 12 minutes or (b, d) 20 minutes. The correlation was done both for (a, b) the TMP-NIT interaction and (c, d) MEC-NIT interaction. The cut-offs for synergy (SIF≤-2) and antagonism (SIF≥2) have been marked with blue and orange doted lines, respectively. The formulas of the corresponding regression lines have been also indicated for each case.

Table S1: Experimental (ExpAUC) and theoretical (ThAUC) area under the curve values of the four biological replicates of each strain tested, indicating also if they correspond to the NIT+MEC or the NIT+TMP antibiotic combination. The average +/- standard deviation of SIF is also indicated (* indicates p<0.05, ** p<0.01, *** p<0.001 and **** p<0.005). FICi values were obtained by CombiANT analyses with, at least, three biological independent assays. Each isolate was classified by synergistic, additive and antagonistic according to the thresholds of SIF and CombiANT. NT means no tested.

Table S2: Normality test of the area under the curve (AUC) of the relative curves in presence of Nitrofurantoin (NIT), trimethoprim (TMP) of both in combination (Exp), as well the theoretical additivity line (Th) for 12 independent assays of the strain 75361 tested for the NIT+TMP combination. The normality test was also run for the SIF values. The statistics analysis was performed with GraphPad Prism, and the five conditions were proved to have a normal distribution by the four tests used.
