# Supplementary material for: Sublethal interaction factor (SIF), a growth-based method to analyze antibiotic combinations at sub-inhibitory concentrations
Source: Microbiol Spectr. 2025 Oct 20;13(12):e01070-25. doi: 10.1128/spectrum.01070-25 (PMC12673930; doi:10.1128/spectrum.01070-25)
Supplement: Supplemental tables — Tables S1 and S2. [file spectrum.01070-25-s0002.pdf]

# Strain	AB combination	Exp_{AUC} #1	Exp_{AUC} #2	Exp_{AUC} #3	Exp_{AUC} #4	Th_{AUC} #1	Th_{AUC} #2	Th_{AUC} #3	Th_{AUC} #4	Exp_{AUC} Vs Th_{AUC}	SIF (+/- SD)	FICi (+/- SD)	Category according SIF	Category according CombiANT (FICi)	Do statistics and SIF match?	Do SIF and FICi match?
4201	NIT+TMP	3,02	3,15	3,1	3,21	7,67	9,2	7,39	10,11	****	-4,63 +/- 1,10	0,49 +/- 0,03	Synergy	Synergy	Yes. Interaction	Yes
62892	NIT+TMP	10,62	11,49	9,61	9,43	10,65	10,91	10,47	10,53	ns	0,00 +/- 0,71	1,96 +/- 1,85	Additive	Additive	Yes. No interaction	Yes
62898	NIT+TMP	7,04	7,06	7,34	6,56	14,21	12,55	12,92	12,8	****	-6,61 +/- 0,67	0,79 +/- 0,24	Synergy	Additive	Yes. Interaction	No
62904	NIT+TMP	7,06	7,7	7,1	6,05	10,76	10	9,6	9,66	****	-3,02 +/- 0,73	0,32 +/- 0,15	Synergy	Synergy	Yes. Interaction	Yes
62942	NIT+TMP	13,28	13,95	14,03	14,75	15,49	15,22	14,41	15,83	ns	-1,23 +/- 0,75	1,08 +/- 0,83	Additive	Additive	Yes. No interaction	Yes
62944	NIT+TMP	11,76	11,5	11,68	11,8	14,66	14,82	14,67	14,72	****	-3,03 +/- 0,19	0,26 +/- 0,02	Synergy	Synergy	Yes. Interaction	Yes
62962	NIT+TMP	7,06	6,59	6,91	6,59	11,31	10,53	10,93	11,04	****	-3,45 +/- 0,22	0,55 +/- 0,38	Synergy	Additive	Yes. Interaction	No
62964	NIT+TMP	9,75	10,41	9,4	10,82	12,39	12,43	12,08	12,17	**	-2,17 +/- 0,61	0,41 +/- 0,25	Synergy	Synergy	Yes. Interaction	Yes
62992	NIT+TMP	6,12	5,59	7,55	6,41	13,34	13,58	14,15	13,49	****	-7,22 +/- 0,58	0,18 +/- 0,10	Synergy	Synergy	Yes. Interaction	Yes
63000	NIT+TMP	5,62	8,76	8,17	9,2	10,29	10,2	12,09	12,97	****	-4,08 +/- 1,37	NT	Synergy	NT	Yes. Interaction	NT
63004	NIT+TMP	3,27	3,14	2,87	2,97	7,85	7,61	7,58	8,59	****	-4,84 +/- 0,52	0,13 +/- 0,01	Synergy	Synergy	Yes. Interaction	Yes
63006	NIT+TMP	6,73	5,83	5,71	6,58	13,22	13,84	14,6	14,92	****	-7,93 +/- 1,02	0,19 +/- 0,07	Synergy	Synergy	Yes. Interaction	Yes
63010	NIT+TMP	6,82	6,66	5,62	5,59	12,93	13,45	7,71	7,36	****	-4,18 +/- 2,62	0,80 +/- 0,35	Synergy	Additive	Yes. Interaction	No
63016	NIT+TMP	9,14	8,84	8,79	7,95	15,69	14,99	15,15	13,71	****	-6,20 +/- 0,34	0,36 +/- 0,40	Synergy	Synergy	Yes. Interaction	Yes
63028	NIT+TMP	8,41	8,27	8,24	7,52	11,22	11,13	11,18	11,5	****	-3,14 +/- 0,55	0,33 +/- 0,35	Synergy	Synergy	Yes. Interaction	Yes
63044	NIT+TMP	3,5	3,77	3,67	3,88	9,84	9,68	9,64	9,87	****	-6,05 +/- 0,20	0,17 +/- 0,08	Synergy	Synergy	Yes. Interaction	Yes
63060	NIT+TMP	10,72	11,1	12,23	13,37	12,93	13,46	13,16	13,44	ns	-0,90 +/- 0,95	0,70 +/- 1,00	Additive	Additive	Yes. No interaction	Yes
63062	NIT+TMP	2,69	2,56	3,21	3,04	9,41	9,53	9,73	9,99	****	-5,86 +/- 0,23	0,12 +/- 0,00	Synergy	Synergy	Yes. Interaction	Yes
63066	NIT+TMP	6,49	5,88	6,67	7,19	10,98	10,23	10,9	10,59	****	-4,11 +/- 0,48	0,12 +/- 0	Synergy	Synergy	Yes. Interaction	Yes
63094	NIT+TMP	5,99	5,58	5,65	6,05	10,52	10,68	10,3	9,82	****	-4,85 +/- 0,44	0,46 +/- 0,10	Synergy	Synergy	Yes. Interaction	Yes
63112	NIT+TMP	10,13	8,19	10,01	10,15	13,51	13,29	13	14,11	****	-3,02 +/- 0,83	0,18 +/- 0,06	Synergy	Synergy	Yes. Interaction	Yes
63128	NIT+TMP	12,42	12,25	10,57	12,25	12,58	12,63	12,16	12,67	ns	-0,11 +/- 0,33	0,81 +/- 0,63	Additive	Additive	Yes. No interaction	Yes
63152	NIT+TMP	3,26	2,52	3,12	3,4	11,22	10,76	10,21	11,05	****	-6,74 +/- 0,44	0,32 +/- 0,42	Synergy	Synergy	Yes. Interaction	Yes
63290	NIT+TMP	6,2	5,23	5,54	5,54	12,81	13,23	13,17	13,27	****	-7,49 +/- 0,60	0,49 +/- 0,31	Synergy	Synergy	Yes. Interaction	Yes
63602	NIT+TMP	9,21	9,13	8,75	9,31	10,5	10,48	10,43	10,13	ns	-1,28 +/- 0,35	0,32 +/- 0,15	Additive	Synergy	Yes. No interaction	No
63626	NIT+TMP	5,45	5,89	5,06	2,7	13,46	13,42	12,93	10,7	****	-6,90 +/- 0,18	0,26 +/- 0,28	Synergy	Synergy	Yes. Interaction	Yes
63642	NIT+TMP	15,67	16,48	17,19	16,58	12,63	12,98	14,19	13,68	****	3,11 +/- 0,26	1,11 +/- 0,54	Antagonism	Additive	Yes. Interaction	No
63662	NIT+TMP	14,41	13,94	11,94	14,19	16,63	15,46	14,7	15,66	*	-1,44 +/- 0,53	0,75 +/- 0,73	Additive	Additive	No	Yes
63664	NIT+TMP	12,19	12,99	12,89	12,78	11,91	11,03	11,68	11,18	ns	1,26 +/- 0,72	0,54 +/- 0,28	Additive	Additive	Yes. No interaction	Yes
63668	NIT+TMP	13,39	12,64	13,66	13,08	14,01	13,86	14,09	14,24	ns	-0,85 +/- 0,38	0,52 +/- 0,15	Additive	Additive	Yes. No interaction	Yes
63670	NIT+TMP	7,66	10,4	10,79	10,66	8,6	11,82	12,18	12,23	ns	-1,33 +/- 0,26	0,59 +/- 0,15	Additive	Additive	Yes. No interaction	Yes
63686	NIT+TMP	10,11	9,47	8,75	10,1	12,38	12,59	12,16	12,39	****	-2,77 +/- 0,57	0,31 +/- 0,24	Synergy	Synergy	Yes. Interaction	Yes
63714	NIT+TMP	12,51	13,21	12,64	12,91	13,08	13,04	12,79	13,17	ns	-0,20 +/- 0,30	0,80 +/- 0,82	Additive	Additive	Yes. No interaction	Yes
63716	NIT+TMP	8,44	8,82	9,82	10,14	11,76	11,88	11,83	11,92	***	-2,53 +/- 0,75	0,34 +/- 0,38	Synergy	Synergy	Yes. Interaction	Yes
63718	NIT+TMP	8,77	8,75	7,63	7,98	11,87	11,99	11,77	12,31	****	-3,70 +/- 0,62	0,30 +/- 0,18	Synergy	Synergy	Yes. Interaction	Yes
63722	NIT+TMP	9,54	8,99	9,18	8,47	13,27	13,1	12,83	13,11	****	-4,03 +/- 0,45	0,37 +/- 0,32	Synergy	Synergy	Yes. Interaction	Yes
63726	NIT+TMP	6,5	6,73	8,63	9,4	11,73	11,23	11,84	12,15	****	-3,27 +/- 1,08	0,15 +/- 0,05	Synergy	Synergy	Yes. Interaction	Yes

# Strain	AB combination	Exp_{AUC} #1	Exp_{AUC} #2	Exp_{AUC} #3	Exp_{AUC} #4	Th_{AUC} #1	Th_{AUC} #2	Th_{AUC} #3	Th_{AUC} #4	Exp_{AUC} Vs Th_{AUC}	SIF (+/- SD)	FICi (+/- SD)	Category according SIF	Category according CombiANT (FICi)	Do statistics and SIF match?	Do SIF and FICi match?
63730	NIT+TMP	3,6	3,03	5,96	3,92	10,64	10,76	11,01	10,47	****	-5,60 +/- 0,98	0,22 +/- 0,06	Synergy	Synergy	Yes. Interaction	Yes
63734	NIT+TMP	3,91	4,49	5,15	4,61	7,51	9,51	10,43	9,92	****	-4,80 +/- 0,81	0,68 +/- 0,22	Synergy	Additive	Yes. Interaction	No
63738	NIT+TMP	16,94	17,23	17,06	17,11	17,1	16,87	17,53	16,48	ns	0,09 +/- 0,49	0,66 +/- 0,28	Additive	Additive	Yes. No interaction	Yes
63746	NIT+TMP	14,38	13,94	14,78	14,27	14,89	14,41	15,39	15	ns	-0,58 +/- 0,12	0,73 +/- 0,19	Additive	Additive	Yes. No interaction	Yes
63758	NIT+TMP	8,17	8,65	8,8	8,38	11,71	12,41	12	12,42	****	-3,63 +/- 0,35	0,73 +/- 0,13	Synergy	Additive	Yes. Interaction	No
63760	NIT+TMP	9,89	8,34	8,45	7,43	15,91	15,43	14,86	14,55	****	-6,66 +/- 0,54	0,44 +/- 0,07	Synergy	Synergy	Yes. Interaction	Yes
63770	NIT+TMP	16,79	16,4	17,21	17,16	19,25	18,49	19,38	19,52	**	-2,26 +/- 0,16	0,48 +/- 0,38	Synergy	Synergy	Yes. Interaction	Yes
63780	NIT+TMP	11,24	11,34	11,83	12,48	16,76	17,12	16,64	16,8	****	-4,26 +/- 0,58	0,24 +/- 0,21	Synergy	Synergy	Yes. Interaction	Yes
63832	NIT+TMP	3,7	4,11	4,67	3,5	7,9	8,11	8,39	7,69	****	-4,02 +/- 0,22	0,49 +/- 0,22	Synergy	Synergy	Yes. Interaction	Yes
63838	NIT+TMP	2,8	3,5	4,71	4,37	10,74	11,75	11,13	11,44	****	-6,33 +/- 0,73	0,40 +/- 0,33	Synergy	Synergy	Yes. Interaction	Yes
63876	NIT+TMP	7,4	8	9,11	6,65	11,8	11,77	11,95	11,73	****	-3,09 +/- 0,83	0,49 +/- 0,02	Synergy	Synergy	Yes. Interaction	Yes
63916	NIT+TMP	12,28	13,33	10,27	10,06	13,42	15,23	13,98	13,66	****	-2,02 +/- 1,11	0,19 +/- 0,08	Synergy	Synergy	Yes. Interaction	Yes
63926	NIT+TMP	5,12	4,83	5,43	4,7	10,41	9,91	10,89	10,65	****	-4,47 +/- 0,29	0,29 +/- 0,07	Synergy	Synergy	Yes. Interaction	Yes
63934	NIT+TMP	5,3	5,58	6,08	7,78	12,78	13,1	14,26	15,1	****	-6,38 +/- 0,33	0,33 +/- 0,31	Synergy	Synergy	Yes. Interaction	Yes
63942	NIT+TMP	2,57	3,8	3,3	3,57	9,59	10,32	10,08	9,96	****	-5,49 +/- 0,35	0,15 +/- 0,05	Synergy	Synergy	Yes. Interaction	Yes
63960	NIT+TMP	5,36	5,39	6,7	6,46	10,66	10,36	11,77	11,35	****	-5,05 +/- 0,17	0,34 +/- 0,09	Synergy	Synergy	Yes. Interaction	Yes
63970	NIT+TMP	7,8	8,69	10,97	8,55	12,45	12,5	12,51	12,36	****	-3,45 +/- 1,32	0,31 +/- 0,05	Synergy	Synergy	Yes. Interaction	Yes
63972	NIT+TMP	11,19	10,53	12,49	12,59	13,27	13,64	13,72	14,04	*	-1,96 +/- 0,84	0,28 +/- 0,14	Additive	Synergy	No	No
63974	NIT+TMP	11,42	11,59	11,94	11,59	11,59	11,19	12,39	12,02	ns	-0,16 +/- 0,40	0,39 +/- 0,38	Additive	Synergy	Yes. No interaction	No
64000	NIT+TMP	14,15	14,45	13,48	14,98	13,24	12,5	10,7	12,7	*	1,89 +/- 0,63	0,90 +/- 1,17	Additive	Additive	No	Yes
64030	NIT+TMP	11,12	9,1	9,82	10,36	13,1	13,08	12,73	13,5	****	-2,38 +/- 0,75	0,53 +/- 0,48	Synergy	Additive	Yes. Interaction	No
64048	NIT+TMP	16,48	16,74	15,57	16,12	16,38	15,87	15,23	15,79	ns	0,41 +/- 0,32	0,59 +/- 0,48	Additive	Additive	Yes. No interaction	Yes
64060	NIT+TMP	3,82	4,15	3,81	3,83	8,27	8,84	8,04	9,05	****	-3,85 +/- 0,38	0,44 +/- 0,24	Synergy	Synergy	Yes. Interaction	Yes
75361	NIT+TMP	7,98	8,11	9,13	8,08	14,548	14,827	14,663	14,106	****	-6,21 +/- 0,53	0,17 +/- 0,07	Synergy	Synergy	Yes. Interaction	Yes
75362	NIT+TMP	11,12	10,8	11,12	11,2	9,262	9,222	9,619	9,296	ns	1,71 +/- 0,19	0,81 +/- 1,08	Additive	Additive	Yes. No interaction	Yes
4201	NIT+MEC	12,29	12,07	12,2	11,69	7,95	7,72	8,43	8,49	****	3,92 +/- 0,55	2,29 +/- 0,28	Antagonism	Additive	Yes. Interaction	No
62892	NIT+MEC	15,44	16,27	15,51	15,87	10,84	10,7	10,67	11,23	****	4,91 +/- 0,45	6,28 +/- 1,40	Antagonism	Antagonism	Yes. Interaction	Yes
62898	NIT+MEC	10,66	13,78	15,87	13,93	5,98	8,97	10,05	8,78	***	5,11 +/- 0,51	1,39 +/- 0,49	Antagonism	Additive	Yes. Interaction	No
62904	NIT+MEC	10,48	10,02	10,33	13,04	7,79	8,49	11,35	10,75	ns	1,37 +/- 1,66	2,02 +/- 0,88	Additive	Additive	Yes. No interaction	Yes
62944	NIT+MEC	16,67	16,57	16,25	16,35	10,61	10,76	11,97	10,37	****	5,53 +/- 0,84	5,90 +/- 1,25	Antagonism	Antagonism	Yes. Interaction	Yes
62962	NIT+MEC	15,66	15,92	16,6	16,67	10,84	9,24	9,79	9,72	****	6,31 +/- 1,00	4,86 +/- 1,77	Antagonism	Antagonism	Yes. Interaction	Yes
62964	NIT+MEC	14,04	13,44	13,48	13,81	10,81	10,41	10,61	10,19	**	3,19 +/- 0,32	4,67 +/- 1,26	Antagonism	Antagonism	Yes. Interaction	Yes
62992	NIT+MEC	8,77	9,57	5,13	6,77	6,43	7,47	7,24	7,15	ns	0,48 +/- 2,12	4,32 +/- 3,45	Additive	Antagonism	Yes. No interaction	No
63006	NIT+MEC	10,74	10,13	11,61	9,82	7,85	7,64	7,27	7,26	**	3,07 +/- 0,86	3,65 +/- 3,34	Antagonism	Additive	Yes. Interaction	No
63010	NIT+MEC	13,68	14,12	13,85	14,21	4,03	7,38	8,15	10,97	****	6,33 +/- 2,65	6,24 +/- 1,55	Antagonism	Antagonism	Yes. Interaction	Yes
63016	NIT+MEC	6,63	6,65	7,1	7,64	7,01	6,64	5,95	9,59	ns	-0,29 +/- 1,28	2,09 +/- 0,87	Additive	Additive	Yes. No interaction	Yes
63028	NIT+MEC	13,86	13,78	13,54	15,74	9,91	9,68	8,59	9,98	****	4,68 +/- 0,84	1,80 +/- 2,35	Antagonism	Additive	Yes. Interaction	No

# Strain	AB combination	Exp_{AUC} #1	Exp_{AUC} #2	Exp_{AUC} #3	Exp_{AUC} #4	Th_{AUC} #1	Th_{AUC} #2	Th_{AUC} #3	Th_{AUC} #4	Exp_{AUC} Vs Th_{AUC}	SIF (+/- SD)	FICi (+/- SD)	Category according SIF	Category according CombiANT (FICi)	Do statistics and SIF match?	Do SIF and FICi match?
63044	NIT+MEC	15,16	15,05	14,85	14,7	9,84	10,25	10,12	10,24	****	4,83 +/- 0,35	7,25 +/- 1,29	Antagonism	Antagonism	Yes. Interaction	Yes
63060	NIT+MEC	13,62	12,89	12,67	12,08	12,06	12,41	12,44	12,07	ns	0,56 +/- 0,68	1,43 +/- 0,77	Additive	Additive	Yes. No interaction	Yes
63062	NIT+MEC	14,45	16,24	14,14	16,64	11,41	12,75	10,98	12,97	**	3,34 +/- 0,29	4,16 +/- 0,89	Antagonism	Antagonism	Yes. Interaction	Yes
63066	NIT+MEC	13,85	13,87	15,22	14,45	11,42	9,82	9,84	11,38	***	3,73 +/- 1,28	6,39 +/- 1,40	Antagonism	Antagonism	Yes. Interaction	Yes
63068	NIT+MEC	23,55	23,41	23,25	23,77	19,4	20,13	19,92	20,54	***	3,49 +/- 0,43	6,64 +/- 1,70	Antagonism	Antagonism	Yes. Interaction	Yes
63094	NIT+MEC	14,74	14,63	15,16	14,88	8,16	7,64	7,98	7,56	****	7,01 +/- 0,32	7,85 +/- 0,24	Antagonism	Antagonism	Yes. Interaction	Yes
63112	NIT+MEC	15,61	16,73	17,03	16,7	12,33	13,88	12,03	13,99	***	3,45 +/- 1,05	4,10 +/- 2,32	Antagonism	Antagonism	Yes. Interaction	Yes
63128	NIT+MEC	11,65	12,3	10,96	12,69	10	10,71	9,15	9,84	ns	1,97 +/- 0,58	2,42 +/- 0,61	Additive	Additive	Yes. No interaction	Yes
63152	NIT+MEC	17,53	18,49	16,77	17,82	15,27	15,68	13,45	14,71	*	2,87 +/- 0,46	4,50 +/-2,92	Antagonism	Antagonism	Yes. Interaction	Yes
63290	NIT+MEC	14,49	15,23	16,41	14,29	9,81	5,18	9,85	4,04	****	7,88 +/- 2,72	8 +/- 0	Antagonism	Antagonism	Yes. Interaction	Yes
63602	NIT+MEC	10,6	11,3	10,09	9,89	8,61	8,92	8,49	8,6	ns	1,82 +/- 0,47	5,50 +/- 1,17	Additive	Antagonism	Yes. No interaction	No
63626	NIT+MEC	14,55	14,81	10,62	15,05	8,91	8,39	7,41	8,92	****	5,35 +/- 1,46	6,26 +/- 1,49	Antagonism	Antagonism	Yes. Interaction	Yes
63642	NIT+MEC	18,21	17,63	17,56	17,68	10,66	10,24	10,23	10,65	****	7,32 +/- 0,21	5,30 +/- 1,13	Antagonism	Antagonism	Yes. Interaction	Yes
63662	NIT+MEC	11,7	12,08	12,68	12,76	8,27	7,57	7,43	8,03	****	4,48 +/- 0,76	5,40 +/- 1,05	Antagonism	Antagonism	Yes. Interaction	Yes
63664	NIT+MEC	15,05	14,41	14,57	13,81	11,71	11,83	11,62	10,22	**	3,11 +/- 0,44	6,60 +/- 2,42	Antagonism	Antagonism	Yes. Interaction	Yes
63668	NIT+MEC	10,41	10,55	10,37	10,16	2,54	2,57	4,49	6,5	****	6,34 +/- 2,03	5,56 +/- 1,33	Antagonism	Antagonism	Yes. Interaction	Yes
63670	NIT+MEC	15,61	15,52	15,75	16,04	15,61	15,52	15,75	16,04	****	4,40 +/- 0,70	8 +/- 0	Antagonism	Antagonism	Yes. Interaction	Yes
63686	NIT+MEC	11,12	11,02	10,98	10,35	11,92	12,51	12,08	11,74	ns	-1,19 +/- 0,31	3,99 +/- 1,91	Additive	Additive	Yes. No interaction	Yes
63714	NIT+MEC	15,21	15,01	14,62	15,32	9,92	9,35	9,21	9,37	****	5,57 +/- 0,29	5,41 +/- 1,73	Antagonism	Antagonism	Yes. Interaction	Yes
63716	NIT+MEC	14,08	15,05	14,08	15,31	7,89	6,38	7,73	5,98	****	7,63 +/- 1,59	6,30 +/- 2,93	Antagonism	Antagonism	Yes. Interaction	Yes
63718	NIT+MEC	10,14	9,68	10,88	10,51	10,64	10,13	10,36	10,26	ns	-0,04 +/- 0,51	2,02 +/- 0,67	Additive	Additive	Yes. No interaction	Yes
63722	NIT+MEC	10,65	10,64	10,37	11,37	10,87	7,25	11,68	6,81	ns	1,60 +/- 2,81	2,95 +/- 3,26	Additive	Additive	Yes. No interaction	Yes
63726	NIT+MEC	17,69	17,86	18,6	17,79	12,69	12,18	12,6	12,65	****	5,45 +/- 0,46	4,10 +/- 2,10	Antagonism	Antagonism	Yes. Interaction	Yes
63730	NIT+MEC	13,36	13,54	13,43	13,99	7,12	5,03	4,96	6,09	****	7,78 +/- 1,06	6,58 +/- 0,92	Antagonism	Antagonism	Yes. Interaction	Yes
63734	NIT+MEC	7,39	7,81	7,03	10,8	10,95	10,31	10,81	11,69	*	-2,68 +/- 1,31	1,10 +/- 0,38	Synergy	Additive	Yes. Interaction	No
63738	NIT+MEC	11,7	11,49	12,21	12,25	5,86	3,87	2,53	6,87	****	7,12 +/- 1,95	5,89 +/- 1,44	Antagonism	Antagonism	Yes. Interaction	Yes
63746	NIT+MEC	8,39	7,39	5,42	4,59	4,53	5,06	6,24	7,34	ns	1,79 +/- 2,38	5,05 +/- 0,60	Additive	Antagonism	Yes. No interaction	No
63758	NIT+MEC	14,99	15,3	14,98	15,33	11,86	11,83	12,07	11,81	**	3,25 +/- 0,28	5,61 +/- 1,04	Antagonism	Antagonism	Yes. Interaction	Yes
63760	NIT+MEC	19,37	19,9	19,61	19,51	18,49	18,6	18,01	19,87	ns	0,85 +/- 0,85	1,68 +/- 0,22	Additive	Additive	Yes. No interaction	Yes
63770	NIT+MEC	17,52	17,68	18,33	17,92	11,33	12,32	13,14	13,09	****	5,39 +/- 0,57	5,84 +/- 2,08	Antagonism	Antagonism	Yes. Interaction	Yes
63792	NIT+MEC	16,09	16,38	16,04	17,08	10,09	11,04	10,61	12,4	****	5,36 +/- 0,54	6,85 +/- 1,20	Antagonism	Antagonism	Yes. Interaction	Yes
63876	NIT+MEC	12,42	11,65	11,33	12,12	11,1	10,95	11,1	10,92	ns	0,85 +/- 0,50	4,47 +/- 2,10	Additive	Antagonism	Yes. No interaction	No
63916	NIT+MEC	9,61	NT	9,54	9,85	8,64	NT	10,18	9,3	ns	0,29 +/- 0,83	2,90 +/- 2,12	Additive	Additive	Yes. No interaction	Yes
63926	NIT+MEC	18,65	18,76	18,49	19,57	15,35	15,58	16,34	16,42	**	2,94 +/- 0,53	8 +/- 0	Antagonism	Antagonism	Yes. Interaction	Yes
63934	NIT+MEC	12,79	16,1	19,07	13,57	13,5	11,67	13,06	13,87	ns	2,35 +/- 3,37	3,82 +/- 3,02	Antagonism	Additive	No	No
63942	NIT+MEC	12,87	13,46	13,47	13,36	12,37	12,75	13,7	11,67	ns	0,67 +/- 0,79	2,10 +/- 1,56	Additive	Additive	Yes. No interaction	Yes
63944	NIT+MEC	11,04	12,32	13,04	12,15	13,62	13,22	12,18	13,45	ns	-0,98 +/- 1,42	3,56 +/- 3,84	Additive	Additive	Yes. No interaction	Yes

# Strain	AB combination	Exp_{AUC} #1	Exp_{AUC} #2	Exp_{AUC} #3	Exp_{AUC} #4	Th_{AUC} #1	Th_{AUC} #2	Th_{AUC} #3	Th_{AUC} #4	Exp_{AUC} Vs Th_{AUC}	SIF (+/- SD)	FICi (+/- SD)	Category according SIF	Category according CombiANT (FICi)	Do statistics and SIF match?	Do SIF and FICi match?
63960	NIT+MEC	18,85	18,14	15,92	17,6	19,39	20,37	14,8	15,96	ns	0,01 +/- 1,74	4,91 +/- 4,19	Additive	Additive	Yes. No interaction	Yes
63970	NIT+MEC	11,39	12,52	13,44	11,17	6,2	8,59	9,5	7,03	****	4,3 +/- 0,60	2,08 +/- 0,70	Antagonism	Additive	Yes. Interaction	No
63972	NIT+MEC	12,43	12,11	12,41	13,7	8,54	6,61	6,57	10,72	****	4,55 +/- 1,34	4,60 +/- 2,63	Antagonism	Antagonism	Yes. Interaction	Yes
63974	NIT+MEC	17,06	16,85	16,04	15,98	10,27	12,09	11,75	11,7	****	7,73 +/- 1,57	8 +/- 0	Antagonism	Antagonism	Yes. Interaction	Yes
64000	NIT+MEC	21,19	21,52	20,98	20,94	19,7	20,23	19,94	19,85	ns	1,22 +/- 0,20	3,07 +/- 1,62	Additive	Additive	Yes. No interaction	Yes
64030	NIT+MEC	17,19	17,77	17,59	17,39	16,57	16,01	16,23	16,53	ns	1,15 +/- 0,51	1,02 +/- 0,25	Additive	Additive	Yes. No interaction	Yes
64048	NIT+MEC	13,07	14,17	13,21	13,87	13,5	13,61	13,56	13,61	ns	-0,01 +/- 0,47	3,12 +/- 2,96	Additive	Additive	Yes. No interaction	Yes
64060	NIT+MEC	13,02	14,83	14,52	14,18	11,94	12,55	12,53	12,45	ns	1,77 +/- 0,51	4,51 +/- 1,20	Additive	Antagonism	Yes. No interaction	No
75361	NIT+MEC	15,78	15,85	15,57	15,16	10,22	9,57	9,47	9,26	****	5,95 +/- 0,30	6,73 +/- 0,95	Antagonism	Antagonism	Yes. Interaction	Yes
75362	NIT+MEC	7,71	9,82	8,39	8,05	4,85	5,19	5,27	4,94	***	3,42 +/- 0,81	2,75 +/- 0,50	Antagonism	Additive	Yes. Interaction	No

Table S1: Experimental (Exp_{AUC}) and theoretical (Th_{AUC}) area under the curve values of the four biological replicates of each strain tested, indicating also if they correspond to the NIT+MEC or the NIT+TMP antibiotic combination. The average +/- standard deviation of SIF is also indicated (* indicates $p<0.05$, ** $p<0.01$, *** $p<0.001$ and **** $p<0.005$). FICi values were obtained by CombiANT analyses with, at least, three biological independent assays. Each isolate was classified by synergistic, additive and antagonistic according to the thresholds of SIF and CombiANT. NT means no tested.

	NIT AUC				TMP UAC				Exp AUC				Th AUC				SIF			
	BR1	BR2	BR3	BR4	BR1	BR2	BR3	BR4	BR1	BR2	BR3	BR4	BR1	BR2	BR3	BR4	BR1	BR2	BR3	BR4
Strain 75361 (NIT+TMP): Independent assay 1	18,01	18,37	18,27	17,66	16,72	17,02	16,32	16,28	7,98	8,11	9,13	8,08	14,55	14,83	14,66	14,11	-6,57	-6,72	-5,54	-6,02
Strain 75361 (NIT+TMP): Independent assay 2	17,73	17,72	18,39	17,94	17,03	16,38	16,23	15,93	9,00	9,95	7,06	7,74	13,82	13,43	13,79	13,44	-4,82	-3,49	-6,73	-5,70
Strain 75361 (NIT+TMP): Independent assay 3	17,80	18,03	17,40	18,28	16,27	16,28	16,05	17,05	7,30	7,28	8,88	7,03	13,62	13,68	13,15	14,17	-6,32	-6,39	-4,28	-7,13
Strain 75361 (NIT+TMP): Independent assay 4	17,16	17,48	17,42	16,72	16,97	17,15	16,89	17,12	7,23	6,78	7,31	6,86	13,57	13,79	13,55	13,33	-6,35	-7,01	-6,24	-6,47
Strain 75361 (NIT+TMP): Independent assay 5	20,19	20,66	20,95	20,91	14,78	14,42	14,61	13,90	7,37	6,97	6,78	7,10	13,06	12,92	13,11	12,73	-5,69	-5,95	-6,32	-5,63
Strain 75361 (NIT+TMP): Independent assay 6	20,44	21,21	20,44	21,37	15,18	15,93	14,96	15,31	7,46	7,83	7,35	7,89	13,55	14,61	13,47	14,06	-6,09	-6,78	-6,12	-6,17
Strain 75361 (NIT+TMP): Independent assay 7	16,97	17,20	17,10	16,85	17,02	16,76	15,80	16,82	6,43	8,54	5,65	6,67	13,39	13,48	12,93	13,54	-6,20	-4,32	-6,46	-6,13
Strain 75361 (NIT+TMP): Independent assay 8	18,58	18,72	19,15	19,17	17,50	16,93	17,01	16,81	8,37	11,49	8,33	8,99	15,10	14,93	14,75	14,83	-5,89	-2,86	-5,57	-5,08
Strain 75361 (NIT+TMP): Independent assay 9	18,19	18,01	17,91	18,27	15,41	15,02	15,93	15,36	5,72	6,11	5,77	6,71	12,75	12,36	12,77	12,95	-7,03	-6,24	-6,99	-6,24
Strain 75361 (NIT+TMP): Independent assay 10	19,93	19,61	19,19	20,10	16,41	15,87	15,80	15,60	11,61	9,65	8,09	9,97	14,61	14,02	13,86	14,09	-3,00	-4,37	-5,78	-4,13
Strain 75361 (NIT+TMP): Independent assay 11	20,21	19,66	19,84	19,45	14,84	14,25	16,38	16,59	11,20	10,48	8,86	10,45	13,61	13,01	14,33	14,47	-2,40	-2,53	-5,48	-4,02
Strain 75361 (NIT+TMP): Independent assay 12	20,16	17,85	19,33	19,68	17,01	15,22	15,88	15,03	11,31	8,37	8,64	9,83	15,15	13,22	13,86	13,72	-3,84	-4,85	-5,21	-3,89
**D'Angostino & Pearson test:** (p-value) normal distributed?	(p-value 0,5410) Yes				(p-value 0,6374) Yes				(p-value 0,5108) Yes				(p-value 0,9383) Yes				(p-value 0,4848) Yes			
**Anderson-Darling test:** (p-value) normal distributed?	(p-value 0,4808) Yes				(p-value 0,5900) Yes				(p-value 0,5960) Yes				(p-value 0,9153) Yes				(p-value 0,2801) Yes			
**Shapiro-Wilk test:** (p-value) normal distributed?	(p-value 0,4538) Yes				(p-value 0,5873) Yes				(p-value 0,6725) Yes				(p-value 0,9924) Yes				(p-value 0,2789) Yes			
**Kolmogorov-Smirnov test:** (p-value) normal distributed?	(p-value >0,1000) Yes				(p-value >0,1000) Yes				(p-value >0,1000) Yes				(p-value >0,1000) Yes				(p-value >0,1000) Yes			

Table S2: Normality test of the area under the curve (AUC) of the relaltive curves in presence of Nitrofurantoin (NIT), trimethoprim (TMP) of both in combination (Exp), as well the theoretical additivity line (Th) for 12 independent assays of the strain 75361 tested for the NIT+TMP combination. The normality test was also run for the SIF values. The statistics analysis was performed with GraphPad Prism, and the five conditions were proved to have a normal distribution by the four tests used.
